# Three-step method for systematic lymphadenectomy in gastric cancer surgery using the ‘curettage and aspiration dissection technique’ with Peng’s multifunctional operative dissector

**DOI:** 10.1186/1477-7819-12-322

**Published:** 2014-10-24

**Authors:** Wenguang Wu, Ping Dong, Xiangsong Wu, Maolan Li, Qichen Ding, Lin Zhang, Jiahua Yang, Hao Weng, Qian Ding, Zhujun Tan, Jianhua Lu, Jun Gu, Yingbin Liu

**Affiliations:** Department of General Surgery and Laboratory of General Surgery, Xinhua Hospital, Affiliated to Shanghai Jiao Tong University, School of Medicine, 1665 Kongjiang Road, Shanghai, 200092 China; Institute of Biliary Tract Disease, Shanghai Jiao Tong University School of Medicine, 1665 Kongjiang Road, Shanghai, 200092 China

**Keywords:** Gastric cancer, Systematic lymphadenectomy, Three-step method, PMOD, CADT

## Abstract

**Background:**

Gastric cancer is one of the most common malignancies and is a leading cause of cancer death worldwide. Surgery is the most effective and successful method of treatment for gastric cancer, and systematic lymph node (LN) dissection is unquestionably the most effective procedure for treating LN metastases of gastric cancer. Systematic lymphadenectomy is the most important part of curative resection, but lymphadenectomy is also the most difficult procedure in gastric cancer surgery. The aim of this study is to report our three-step method for lymphadenectomy in gastric cancer.

**Methods:**

In this study, the lymph node stations and groups were defined according to the 13th edition of the Japanese Classification for Gastric Carcinoma. The authors’ novel, simplified method consists of three steps: (1) the Kocher maneuver and dissection of the greater omentum together with the anterior sheet of the mesocolon, (2) dissection of the lesser omentum, and (3) lymphadenectomy following the main vessels. We primarily used Peng’s multifunctional operative dissector, which combines four different functions (cutting, separating, aspirating and coagulating). Our systematic lymphadenectomy included three steps, and the main procedure started from right to left and in the caudal to cranial direction.

**Results:**

A total of 830 consecutive patients underwent our three-step-method systematic lymphadenectomy in advanced gastric cancer surgery. The mean operation time was 146 minutes, and the mean blood loss was 248 ml. The median postoperative hospital stay was 10.9 ± 4.8 days. The median number of examined LN was 31.6 (range 17 to 72) per patient, and the median number of metastatic LN was 5.6 (range 0 to 42) per patient. The overall incidence of postoperative complications was 10.6%, and the rate of hospital death was 0.9%. The overall three-year survival rate was 52.6%.

**Conclusions:**

Our three-step method for lymphadenectomy is easy to perform and is a useful procedure for gastric cancer surgery.

## Background

**Figure 1:**
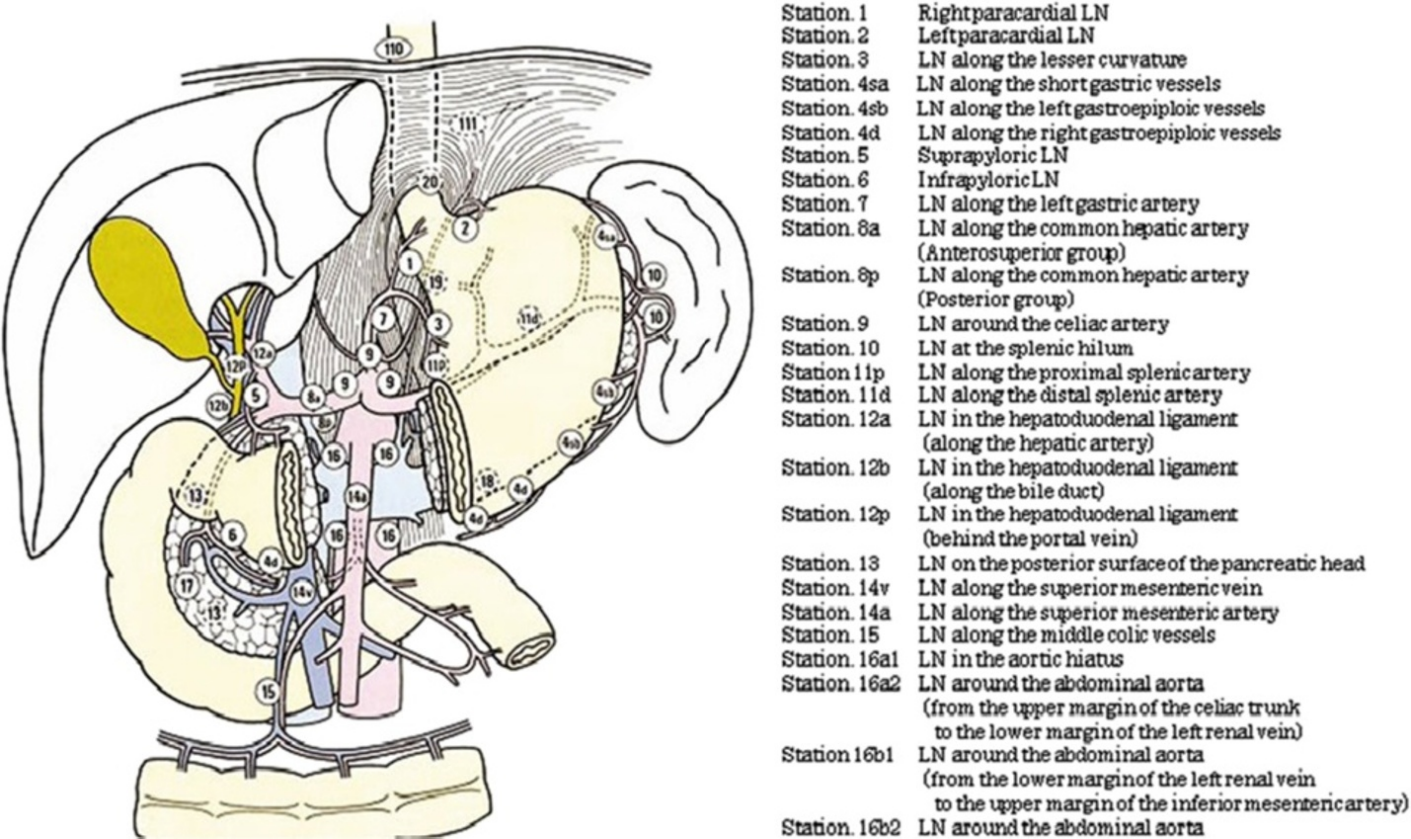
**Lymph node station numbers were reproduced from the Japanese classification of gastric carcinoma 2nd English edition with permission from original authors [**[1]**].** LN, Lymph node.

Gastric cancer is one of the most common malignancies and a leading cause of cancer death worldwide. Surgery is the most effective and successful method of treatment for gastric cancer, and systematic lymph node (LN) dissection is unquestionably the most effective procedure for treating LN metastases of gastric cancer. Systematic lymphadenectomy is the most important part of curative resection, but lymphadenectomy is also the most difficult procedure in gastric cancer surgery. Knowledge regarding LN staging is mandatory for understanding LN dissection. The complex LNs of the stomach have been organized by a useful classification system described in the Japanese Classification for Gastric Carcinoma (JCGC) [1]. The JCGC staging system was designed to describe the anatomic locations of the nodes removed during gastrectomy. According to this classification system, 16 different LN stations surrounding the stomach were identified (Figure 1). The purpose of this study is to introduce a novel procedure for safe and simple lymphadenectomy in gastric cancer surgery. Our three-step method for lymphadenectomy routinely removes all above mentioned nodes except the para-aortic node (station number 16); however, the details regarding which node groups should be removed in different patients depend on the site of the tumor and the level of dissection required [2].

## Methods

**Figure 2:**
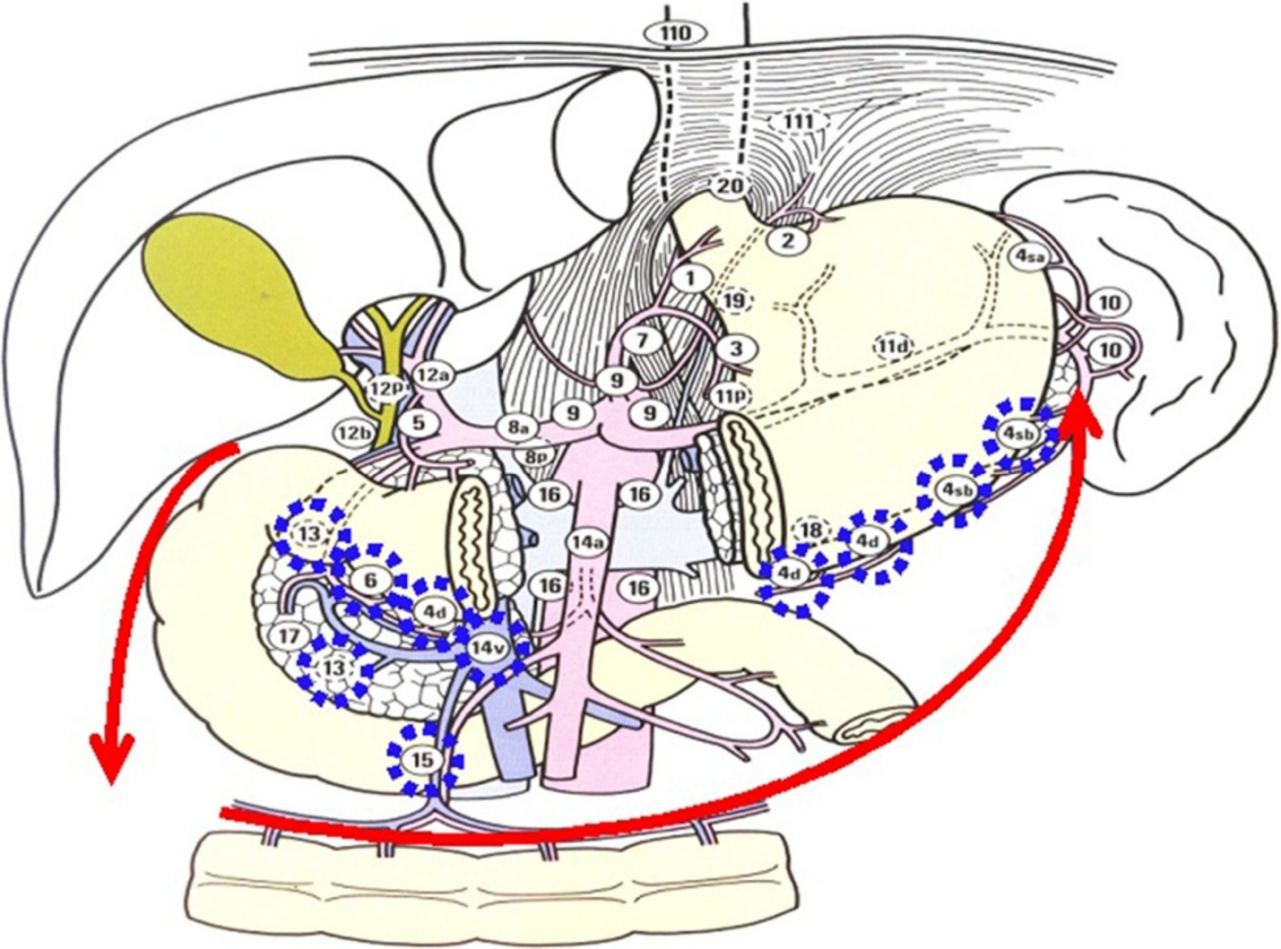
**The node stations to remove in step one, including Nodal station numbers 13, 15, 14v, 6, 4d, and 4sb.** A Kocher maneuver and dissection of the greater omentum together with the anterior sheet of the mesocolon from right to left (arrows). Lymph node station numbers were reproduced from the Japanese classification of gastric carcinoma 2nd English edition with permission from original authors.

**Figure 3:**
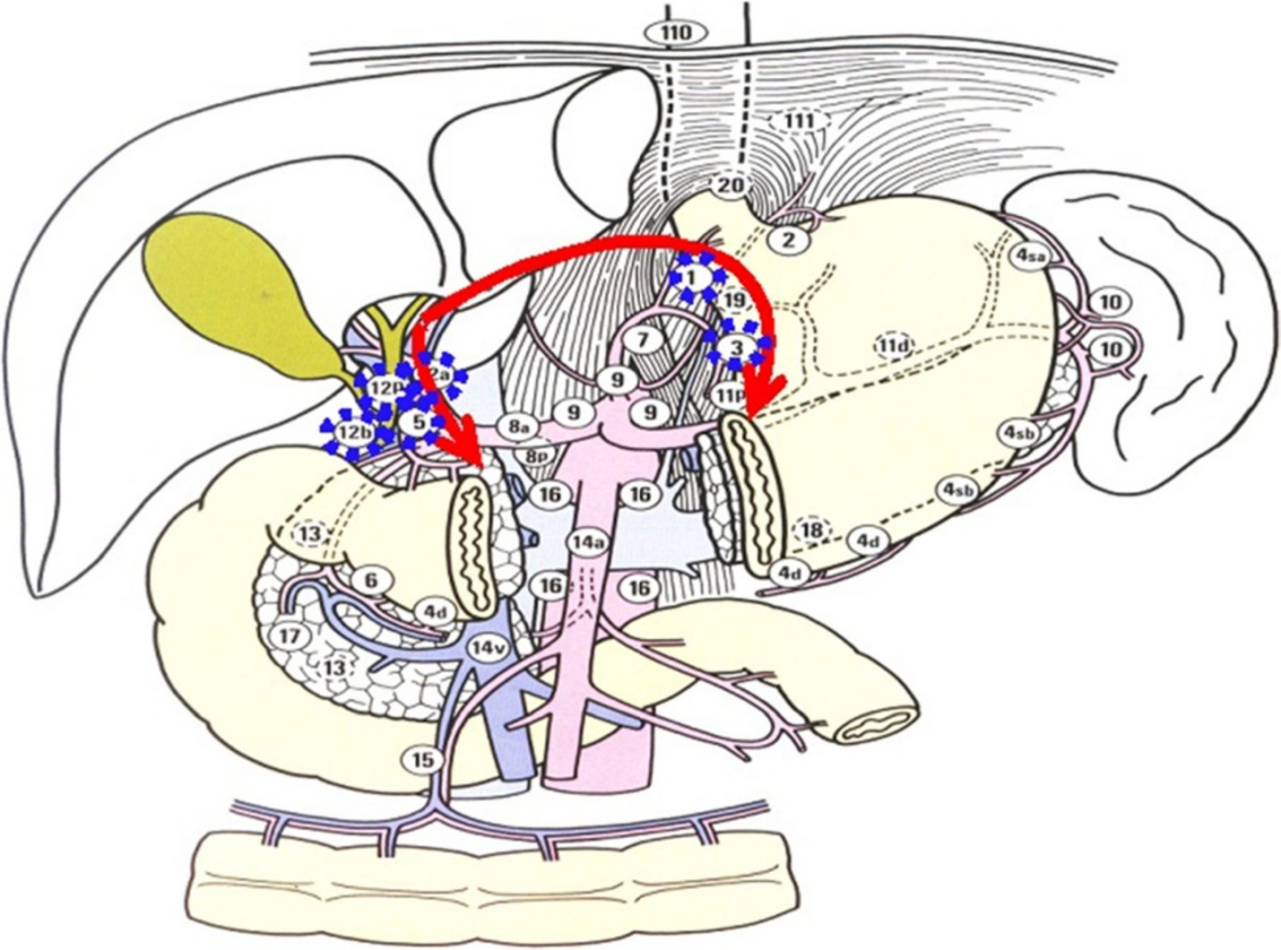
**The node stations to remove in step two, including Nodal station numbers 12, 5, 1, and 3.** The lymphadenectomy on hepatoduodenal ligament and dissection of the lesser omentum (arrows). Lymph node station numbers were reproduced from the Japanese classification of gastric carcinoma 2nd English edition with permission from original authors.

**Figure 4:**
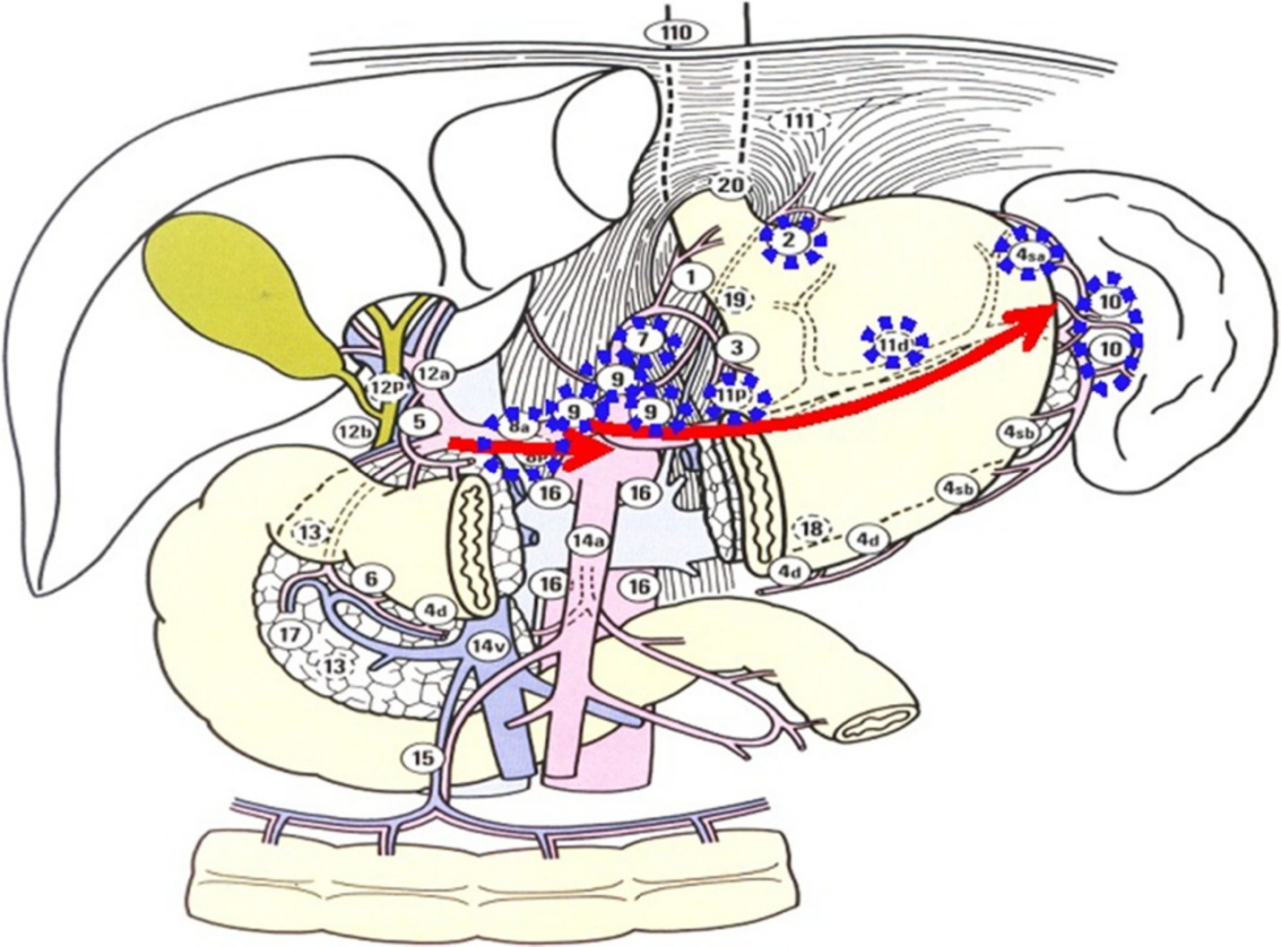
**The node stations to remove in step three, including Nodal station numbers 8, 7, 9, 11p, 11d, 10, 4sa, and 2.** Lymphadenectomy along the main vessels (arrows). Lymph node station numbers were reproduced from the Japanese classification of gastric carcinoma 2nd English edition with permission from original authors.

This study was approved by the ethical institutional board and was performed at the Department of General Surgery, Xinhua Hospital, School of Medicine, Shanghai Jiaotong University. The patients had consented to the publication of the images in this manuscript. In this study, the LN stations and groups were defined according to the 13th edition of the Japanese Classification for Gastric Carcinoma. Our novel, simplified method consists of three steps: (1) a Kocher maneuver and dissection of the greater omentum together with the anterior sheet of the mesocolon (Figure 2), (2) dissection of the lesser omentum (Figure 3), and (3) lymphadenectomy along the main vessels (Figure 4). We primarily used Peng’s multifunctional operative dissector (PMOD) with the ‘curettage and aspiration dissection technique’ (CADT) [3–6], which combines four different functions: cutting, separating, aspirating, and coagulating. The lymphadenectomy included three steps, and the main procedure was initiated from right to left and in the caudal to cranial direction [7].

### Step one

**Figure 5:**
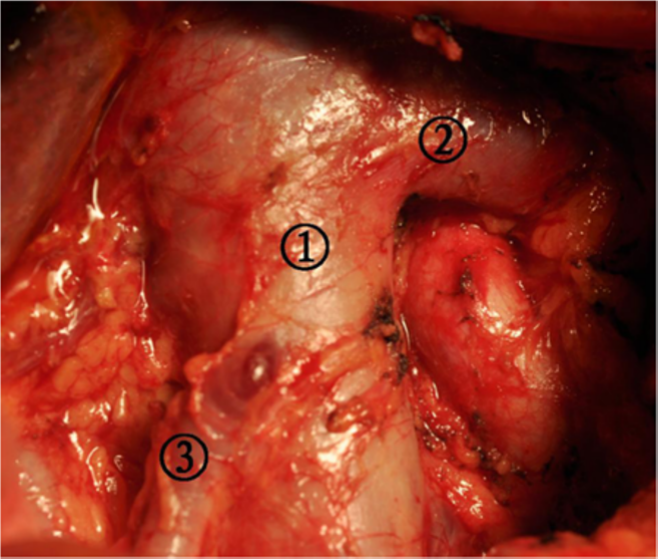
**Kocher maneuver was performed to expose the duodenum, the head of the pancreas, and the inferior vena cava to exclude undetected metastases at station number 13.** (1) Inferior vena cava, (2) Left renal vein, and (3) The right testicular vein.

**Figure 6:**
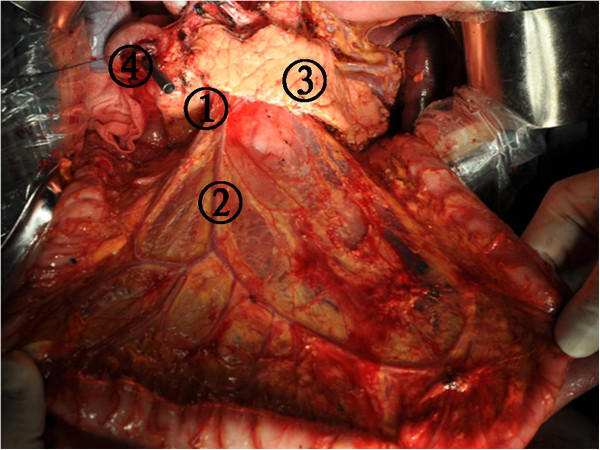
**The greater omentum and the anterior sheet of the mesocolon and pancreatic capsule were dissected from right to left.** (1) The dissection nodal station number 14, (2) The dissection nodal station number 15, (3) Pancreas, and (4) Stump of duodenum.

Step one consisted of the Kocher maneuver and the dissection of the greater omentum together with the anterior sheet of the mesocolon. The right Gerota’s membrane and perirenal fat were lifted to reveal the right renal blood vessels and the right testicular or ovarian blood vessels. Next, a Kocher maneuver was performed to expose the duodenum, the head of the pancreas, and the inferior vena cava to exclude undetected metastases at station number 13 (Figure 5). The dissection was initiated at the duodenal membrane after the Kocher maneuver was completed. Subsequently, the greater omentum and the anterior sheet of the mesocolon and pancreatic capsule were dissected from right to left (Figure 6). The transverse colon was pulled caudally by the first assistant, and the surgeon lifted the greater omentum with the left hand so that the anterior sheet could be easily dissected from the underlying tissue between the hepatic flexure and the splenic flexure using PMOD in the right hand. The greater omentum was dissected from the transverse colon together with the anterior sheet of the mesocolon. At the midpoint of the colon, where the interstitial space between the anterior lobe and the posterior lobe of the mesocolon is narrow, we used the CADT together with electrical coagulation and cutting to widen this space by pushing aside and separating the tissue. As we dissected this sheet, the right accessory colic vein was located and followed cranially to the point where it joins Henle’s surgical trunk. The origin of the right gastroepiploic vein was then identified, ligated, and divided at its origin. This dissection was performed from the inferior to the superior border of the pancreas and from the middle of the pancreas toward the duodenum until the gastroduodenal artery was found. This artery was then followed caudally to the right gastroepiploic artery, which was ligated and divided at its origin. Nodal station numbers 13, 15, 14v, 6, 4d, and 4sb were dissected in the first step.

### Step two

**Figure 7:**
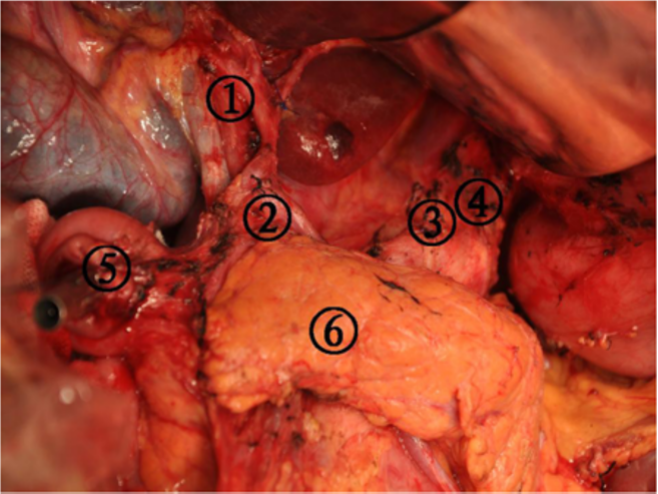
**A dissection was performed from the proper hepatic artery to the common hepatic artery and from the common hepatic artery to the left gastric artery until the celiac artery was located.** (1) The dissection nodal station number 12, (2) The dissection nodal station number 5, (3) The dissection nodal station number 7, (4) The dissection nodal station number 9, (5) Stump of duodenum, and (6) Pancreas.

**Figure 8:**
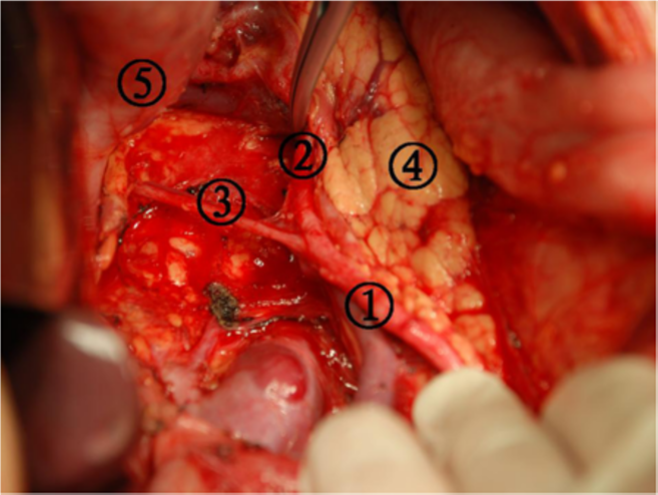
**The origin of the splenic artery was located, and the dissection was performed along the splenic artery until the secondary branches of the splenic pedicle were located.** (1) The dissection nodal station number 11p, (2) The dissection nodal station number 11d, (3) Posterior gastric artery, (4) Pancreas, and (5) Stomach.

Step two consisted of the dissection of the lesser omentum. Tissue in the hepatoduodenal ligament was dissected along the proper hepatic artery from the liver toward the duodenum. We divided the right gastric artery at its origin after the proper hepatic artery was located to eliminate the possibility of damage and to obtain clear visualization (Figure 7). The lesser omentum was then divided just below the liver by using electrical coagulation with PMOD from the right side of the proper hepatic artery to the left side of the esophageal artery. This division was followed caudally along the lesser curvature, if required. During this step, since the space was narrow, we used PMOD to push the tissue aside and open the space. The operator finished this step alone in order to obtain clear surgical visualization and minimize operation time (Figure 8). Nodal station numbers 12, 5, 1, and 3 were dissected in the second step.

### Step three

**Figure 9:**
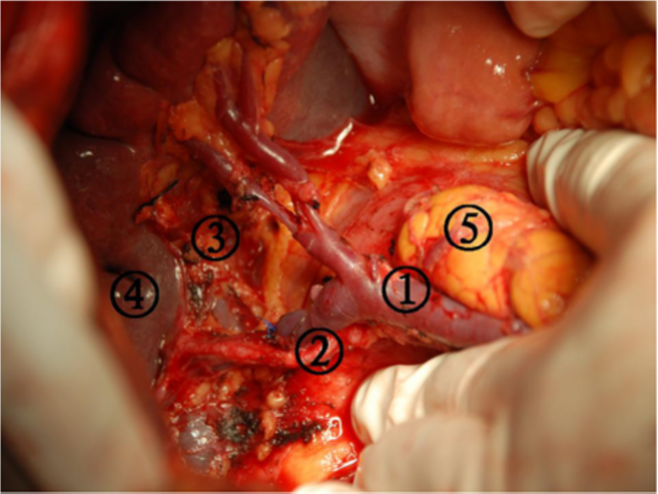
**The secondary branches of the splenic pedicle was exposed in the dissection nodal station numbers 10 and 11.** (1) Splenic vein, (2) Splenic artery, (3) The dissection nodal station number 11p, (4) Spleen, and (5) Pancreas tail.

Step three consisted of a lymphadenectomy along the main vessels. A dissection was performed from the proper hepatic artery to the common hepatic artery and from the common hepatic artery to the left gastric artery until the celiac artery was located (Figure 7). The dissection was initiated at the proper hepatic artery, and lymphadenectomy was performed along the main arteries. Next, the origin of the splenic artery was located, and the dissection was performed along the splenic artery until the secondary branches of the splenic pedicle were located (Figures 8 and 9) [8]. If the tumor involved the upper or middle third of the stomach, the dissection was performed up to the left side of the cardia in this step. Nodal station numbers 8, 7, 9, 11p, 11d, 10, 4sa, and 2 were dissected in the final step.

## Results

A total of 830 consecutive patients underwent our three-step method systematic lymphadenectomy in advanced gastric cancer surgery using the curettage and aspiration dissection technique with Peng’s multifunctional operative dissector between February 2006 and December 2009. The main surgery procedure was performed from right to left and in the caudal to cranial direction. The mean operation time was 146 minutes, and the mean blood loss was 248 ml. The median postoperative hospital stay was 10.9 ± 4.8 days. The median number of examined LN was 31.6 (range 17 to 72) per patient, and the median number of metastatic LN was 5.6 (range 0 to 42) per patient. The overall incidence of postoperative complications was 10.6%, and the rate of hospital death was 0.9%. The the overall three-year survival rate was 52.6%.

## Discussion

The two main staging systems for gastric cancer are the TNM staging system of the Union for International Cancer Control (UICC) and the JCGC of the Japanese Gastric Cancer Association (JGCA). In the TNM system, a number-based system is used for N-staging, and this system allows for simple and accurate prognostic stratification. Comparative studies have shown that the TNM system has greater prognostic power than the Japanese classification system [9, 10]. However, the TNM system provides no treatment guidance and should primarily be used as a guide for prognosis. In contrast, the Japanese classification system has been designed as a comprehensive guide to treatment; its anatomy-based N-staging system was established on the basis of an analysis of lymphadenectomy effectiveness. Therefore, it provides direct surgical guidance for systematic lymphadenectomy. Recently, LN metastasis staging in gastric cancer was revised in both the seventh edition of the UICC and the 14th edition of the JGCA staging systems such that staging now depends solely on the number of metastatic nodes uncovered [11, 12]. In the new UICC and JGCA systems, patients with one to two metastatic LNs are classified as N1, those with three to six metastatic LNs are classified as N2, and those with seven or more metastatic LNs are classified as N3. In our clinical experience, we believe that the N-number classification system of LN metastases from gastric cancer has greater prognostic power, whereas the N-site classification system is a useful guide for surgeons performing systematic lymphadenectomy.

**Figure 10:**
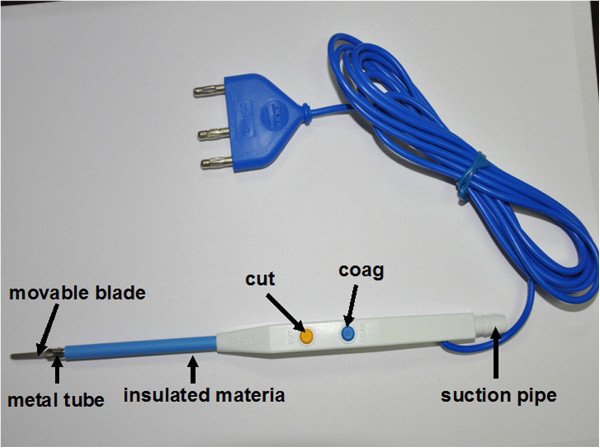
**Peng’s Multifunction Operative Dissector (PMOD).** Hangzhou Shuyou Medical Instrument Co, Hangzhou, Zhejiang, China, FDA 510(K) number K040780. PMOD is a special instrument that combines four different functions: cutting, separating, aspirating, and coagulating.

In 1990, Professor Shuyou Peng developed a versatile electrosurgical instrument called Peng’s multifunctional operative dissector (PMOD; Hangzhou Shuyou Medical Instrument Co., Hangzhou, Zhejiang, China, FDA 510(K) number K040780) for liver parenchymal transection (Figure 10) [13]. PMOD is a special instrument that combines four different functions: cutting, separating, aspirating, and coagulating. Thus, PMOD eliminates the necessity of frequently changing surgical instruments, which reduces operating times and bleeding and improves the quality of surgery. Therefore, this dissection technique is named the curettage and aspiration dissection technique (CADT). When performing surgical dissection with PMOD, the surgeon and his or her assistant must maintain the tension of the tissue being dissected by pulling it from the opposite direction. Note that the electrical cutter should never be turned on when curettage is performed, but the suction should always be on. The best aspect of PMOD is that it can delineate all the vessels and the ductal system; thus, PMOD is more suitable for lymphadenectomy in gastric cancer surgery and greatly improves the quality of the operation. Use of the CADT saves operating time, reduces blood loss, and keeps the surgical field clear by eliminating the need to frequently change surgical instruments. Additionally, suction is maintained during the surgery. Blood, the plume produced by electrical coagulation, and tissue are aspirated immediately.

## Conclusions

Our three-step method for systematic lymphadenectomy is easy to perform and is a useful procedure for gastric cancer surgery.

## Abbreviations

CADT: Curettage and aspiration dissection technique
JCGC: Japanese Classification for Gastric Carcinoma
JGCA: Japanese Gastric Cancer Association
UICC: Union for International Cancer Control
LN: lymph node
PMOD: Peng’s multifunctional operative dissector.
